# Probiotic and synbiotic therapy in critical illness: a systematic review and meta-analysis

**DOI:** 10.1186/s13054-016-1434-y

**Published:** 2016-08-19

**Authors:** William Manzanares, Margot Lemieux, Pascal L. Langlois, Paul E. Wischmeyer

**Affiliations:** 1 Department of Critical Care, Intensive Care Unit, Hospital de Clínicas (University Hospital), Faculty of Medicine, Universidad de la República (UdelaR), Italia Av, 14th Floor, 11.600, Montevideo, Uruguay; 2 Clinical Evaluation Research Unit. Angada 4, Kingston General Hospital, 76 Stuart Street, Kingston, ON K7L 2V7 Canada; 3 Département de Anesthésie et de Réanimation, Faculté de Médecine et des Sciences de la Santé, Université de Sherbrooke, Centre Hospitalier Universitaire de Sherbrooke–Hôpital Fleurimont, Pièce 3610 3001, 12e Avenue Nord, Sherbrooke, QC J1H 5N4 Canada; 4 Department of Anesthesiology and Pediatrics (Nutrition Section), University of Colorado, School of Medicine, 12700 E. 19th Ave., RC2 P15-7120, Box 8602, Aurora, CO 80045 USA

**Keywords:** Probiotics, Synbiotics, Critical care, Infections, Ventilator-associated pneumonia, Systematic review

## Abstract

**Background:**

Critical illness is characterized by a loss of commensal flora and an overgrowth of potentially pathogenic bacteria, leading to a high susceptibility to nosocomial infections. Probiotics are living non-pathogenic microorganisms, which may protect the gut barrier, attenuate pathogen overgrowth, decrease bacterial translocation and prevent infection. The purpose of this updated systematic review is to evaluate the overall efficacy of probiotics and synbiotic mixtures on clinical outcomes in critical illness.

**Methods:**

Computerized databases from 1980 to 2016 were searched. Randomized controlled trials (RCT) evaluating clinical outcomes associated with probiotic therapy as a single strategy or in combination with prebiotic fiber (synbiotics). Overall number of new infections was the primary outcome; secondary outcomes included mortality, ICU and hospital length of stay (LOS), and diarrhea. Subgroup analyses were performed to elucidate the role of other key factors such as probiotic type and patient mortality risk on the effect of probiotics on outcomes.

**Results:**

Thirty trials that enrolled 2972 patients were identified for analysis. Probiotics were associated with a significant reduction in infections (risk ratio 0.80, 95 % confidence interval (CI) 0.68, 0.95, *P* = 0.009; heterogeneity *I*^2^ = 36 %, *P* = 0.09). Further, a significant reduction in the incidence of ventilator-associated pneumonia (VAP) was found (risk ratio 0.74, 95 % CI 0.61, 0. 90, *P* = 0.002; *I*^2^ = 19 %). No effect on mortality, LOS or diarrhea was observed. Subgroup analysis indicated that the greatest improvement in the outcome of infections was in critically ill patients receiving probiotics alone versus synbiotic mixtures, although limited synbiotic trial data currently exists.

**Conclusion:**

Probiotics show promise in reducing infections, including VAP in critical illness. Currently, clinical heterogeneity and potential publication bias reduce strong clinical recommendations and indicate further high quality clinical trials are needed to conclusively prove these benefits.

## Background

Critical illness is characterized by a loss of commensal flora and an overgrowth of potentially pathogenic bacteria, leading to a high susceptibility to acquired nosocomial infections [1, 2]. Further, sepsis following infection is still a leading cause of death worldwide [3]. The U.S. Centers for Disease Control indicates death rates from critical illness/sepsis have increased at a rate greater than any other common cause of mortality in the last year for which data were available [4]. Thus, therapies to reduce the risk and incidence of infection and sepsis in critical illness are urgently needed.

According to the World Health Organization and the Food and Agriculture Organization, probiotics are living non-pathogenic microorganisms, which have demonstrated well-documented beneficial health effects administered in optimum amounts in the prevention and treatment of several disease states [5]. So far, several mechanisms by which probiotics may exert beneficial effects have been described, including modification of the gut flora by inducing host cell antimicrobial peptides, release of antimicrobial factors, suppression of the immune cell proliferation, stimulation of mucus and IgA production, anti-oxidative activity, inhibition of epithelial cell nuclear factor kappa B activation, and other potentially vital gut epithelial barrier protective effects [6–8]. As the gut is hypothesized to play a central role in the progression of critical illness, sepsis and multiple organ dysfunction syndrome [9], maintenance of the gut barrier and a healthy gut microbiome, potentially via reintroduction of commensal bacteria (probiotic therapy), may be essential to optimizing outcomes in critically ill patients.

According to current literature, the efficacy of probiotics in the prevention of infectious complications has been extensively evaluated in many animal studies and clinical trials in heterogenous intensive care unit (ICU) patient populations. These studies suggest that probiotics may reduce the incidence of infection, particularly ventilator-associated pneumonia (VAP) [10], which is a common serious complication in intubated, mechanically ventilated patients [11]. Nonetheless, the effect of probiotics on the prevention of VAP still remains controversial and inconclusive [12–17]. In fact, its effect depends on the patient population and the probiotic strain studied. Despite the outcome benefits of probiotics therapy, recent guidelines have been unable to make a definitive recommendation for the routine use of probiotics in ICU patients. To date, these guidelines have suggested the use of probiotic therapy in select medical and surgical patient populations in whom trials have documented safety and clinical benefits [18, 19].

Over the last few years, several systematic reviews and meta-analyses have evaluated the effects of probiotics in critically ill patients [12–17]. In 2012, after aggregating 11 trials that reported on infections [14], we demonstrated that probiotics may reduce infections, including the incidence of VAP, although the effect on VAP was not statistically significant given the available data. Moreover, probiotics were associated with a trend toward reduced ICU mortality, but did not influence hospital mortality. Since our last systematic review and meta-analyses, seven new trials of probiotic therapy have been published [20–26]. Further, to date, no recent meta-analysis has examined the effect of probiotic versus synbiotic (probiotic and prebiotic fiber) therapy. Finally, a Canadian survey [27] on the use of probiotics as a prophylactic strategy for VAP showed that most Canadian ICU pharmacists have used probiotics at least once, although routine use is considered controversial and considerable practice variability exists. Thus, any increased understanding that the newly published trials can yield will be vital to clarifying clinical probiotic use in the ICU and areas in need of future research focus.

Therefore, as probiotic use in the ICU remains widespread and controversial, current guidelines are not conclusive, and with a significant number of new trials of probiotic use published recently we conducted a comprehensive systematic review and meta-analysis of probiotic and synbiotic use in critically ill patients. Our aim was to elucidate the overall efficacy of probiotics, as a single strategy or in combination with fiber therapy (synbiotics) on relevant clinical outcomes, particularly infection and VAP, in adult critically ill patients.

## Methods

### Search strategy and study identification

A literature search was conducted in MEDLINE, Embase, CINAHL, the Cochrane Central Register of Controlled Trials and the Cochrane Database of Systematic Reviews to identify all relevant randomized controlled trials (RCTs) published between 1980 and April 2016. The literature search used broad search terms containing “randomized,” “clinical trial,” “nutrition support,” “enteral nutrition”, “probiotics,” and “synbiotics”. No language restrictions were applied. Personal files and reference lists of relevant review articles were also reviewed.

### Eligibility criteria

We included trials with the following characteristics:

1. Type of study: randomized controlled parallel group trials
2. Population: adult (≥18 years of age) critically ill patients. If the study population was unclear, we considered a mortality rate higher than 5 % in the control group to be consistent with critical illness
3. Intervention: Probiotics alone or associated with prebiotics (synbiotics) compared to a placebo
4. Outcomes: pre-specified clinical outcomes in ICU patients such as infectious complications, VAP, mortality, ICU and hospital length of stay (LOS), and diarrhea

We excluded trials that reported only nutrition, biochemical, metabolic, or immunologic outcomes. Data published in abstract form were included only if additional information about the study design was obtained from the authors. The methodological quality of the included trials was assessed in duplicate by two reviewers independently using a data abstraction form with a scoring system from 0 to 14 according to the following criteria:

1. The extent to which randomization was concealed
2. Blinding
3. Analysis based on the intention-to-treat (ITT) principle
4. Comparability of groups at baseline
5. Extent of follow up
6. Description of treatment protocol
7. Co-interventions
8. Definition of clinical outcomes

Consensus between both reviewers on the individual scores of each of the categories was obtained. We attempted to contact the authors of included studies and requested additional information not contained in published articles. We designated studies as level I if all of the following criteria were fulfilled: concealed randomization, blinded outcome adjudication and an ITT analysis, all which are the strongest methodological tools to reduce bias. A study was considered as level II if any one of the above-described characteristics were unfulfilled.

### Data synthesis

All analyses, except the test for asymmetry, were conducted using RevMan 5.3 (Cochrane IMS, Oxford, UK) with a random effects model. We combined data from all trials to estimate the overall weighted mean difference (WMD) with 95 % confidence intervals for LOS data the pooled risk ratio (RR) with 95 % confidence intervals (CIs) for the incidence of infections and mortality, and diarrhea. WMDs were estimated by the inverse variance approach and pooled RRs were calculated using the Mantel-Haenszel estimator. The random effects model of DerSimonian and Laird was used to estimate variances for the Mantel-Haenszel and inverse variance estimators [28]. RRs were undefined and excluded for studies with no event in either arm. Heterogeneity was tested by a weighted Mantel-Haenszel χ^2^ test and quantified by the *I*^2^ statistic as implemented in RevMan. Differences between subgroups were analyzed using the test of subgroup differences described by Deeks et al., and the results expressed using the *P* values. We considered *P* <0.05 to be statistically significant and *P* <0.10 as an indicator of trends. Funnel plots were used to assess the possibility of publication bias and the Egger regression test was used to measure funnel plot asymmetry [29].

### Clinical outcomes

Overall infections were the primary outcome for this meta-analysis. Secondary outcomes were VAP, mortality, ICU and hospital LOS, and finally diarrhea. We used definitions of infections as defined by the authors in their original articles. From all trials, we combined hospital mortality where reported. Mortality specified at either 28 days or 90 days was not considered as ICU or hospital mortality, respectively. Nonetheless, if the mortality time frame was not specified as either ICU or hospital, it was presumed to be the later.

### Subgroup analysis

We utilized predefined subgroup analyses to assess a number of possible influences on the effect of probiotic supplementation on clinical outcomes, and thus to explore the possible causes of heterogeneity. On the basis that the higher the daily dose the greater the effect, we first examined trials that administered a high dose of probiotics defined as >5 × 10^9^ colony-forming units (CFU)/day vs. lower dose probiotics defined as <5 × 10^9^ CFU/day. Second, we compared the results of RCTs that administered *Lactobacillus plantarum* as probiotic therapy vs. no *L. Plantarum*, and compared trials using *Lactobacillus rhamnosus* strain GG (LGG) vs. those administering other non-LGG strains.

Moreover, based on a larger treatment effect in those more seriously ill patients with higher risk of death, we compared studies including patients with higher mortality vs. lower mortality. Mortality was considered to be high or low based on whether it was greater or less than the median control group mortality of all the trials. Trials of higher quality, defined as those with a methodological score equal to or higher than the median quality score, may demonstrate a lower treatment effect.

## Results

### Study identification and selection

A total of 79 relevant citations were identified from the search of computerized bibliographic databases and a review of reference lists from related articles. Of these, we excluded 49 due to the following reasons: 21 trials did not include ICU patients (mostly surgical patients); 12 articles were systematic reviews and meta-analyses; 4 trials were published as an abstract and we were unable to obtain the data from the authors to complete our data abstraction process; 5 articles were duplicates of included trials; 3 studies did not evaluate clinical outcomes; 2 trials tested multiple interventions; 1 study was not a RCT, and finally 1 study administered probiotics as oral swabs.

Finally, 30 RCTs [10, 20–26, 30–51] met our inclusion criteria and were included, covering a total of 2972 patients (see Tables 1 and 2). The reviewers reached 100 % agreement on the inclusion of the trials. The mean methodological score of all trials was 9, whereas the median value was 9.5 on a maximum of 14 (range 5–13). Randomization was concealed in 9/30 trials (30 %), ITT analysis was performed in 18/30 trials (60 %), and double blinding was done in 20/30 of the studies (67 %). There were five level-I studies and 25 level-II studies. The details of the methodological quality of the individual trials are shown in Table 1.

**Table 1:** Randomized studies evaluating probiotics in critically ill patients

	Study	Population	Methods score	Type of probiotic/intervention		
				Delivery vehicle	Intervention/dose/duration	Control
1	Tempe 1983 [30]	ICU patients n = 40	C.Random: yes ITT: yes Blinding: double Score: 10 Viability (intervention): NR	EN tube	EN (unknown) + Ultra-Levure (*Saccharomyces boulardii*), 10^10^/1 L solution for 11–21 days	EN (unknown) + placebo (sterile solution)
2	Schlotterer 1987 [31]	Patients with burns n = 18	C.Random: no ITT: no Blinding: double Score: 8 Viability (intervention): NR	NG tube	EN (Polydiet or Nutrigil) + *Saccharomyces boulardi* 500 mg QID for 8-28 days	EN (Polydiet or Nutrigil) + placebo
3	Heimburger 1994 [32]	Mixed ICU patients: 83 % received antibiotics n = 62	C.Random: no ITT: no Blinding: double Score: 9 Viability (intervention): NR	EN tube	EN (standard) + 1 g of Lactinex (*Lactobacillus acidophilus* and *Lactobaccilus bulgaricus*) 2 × 10^6^TID for 5–10 days	EN (standard) + placebo (0.5 g dextrose + 0.5 g lactose)
4	Bleichner 1997 [33]	Mixed ICU patients n = 128	C.Random: not sure ITT: yes Blinding: double Score: 13 Viability (intervention): NR	EN tube	EN (unknown) + *Saccharomyces boulardii* 500 mg QID for 21 days or until EN stopped	EN (unknown) + placebo (powder)
5	Kecskes 2003 [34]	ICU patients on antibiotics n = 45	C.Random: no ITT: no Blinding: double Score: 8 Viability (intervention): yes	NJ tube	EN (Nutrison fiber) + fermented oatmeal formula with *Lactobacillus plantarum* 299 10 ^9^ BID and fiber for 7 days	EN (Nutrison fiber) + heat-killed *Lactobacillus plantarum* 299 BID + fiber (non-viable)
6	Jain 2004 [36]	ICU patients n = 90	C.Random: no ITT: yes Blinding: double Score: 10 Viability (intervention): NR	Oral or NG tube	EN or PN + Trevis™ 1 capsule TID + 7.5 g Raftilose (oligofructose) BID until hospital discharge	EN or PN + placebo (powdered sucrose capsules)
7	Lu 2004 [35]	Patients with burns n = 40	C.Random: no ITT: yes Blinding: double Score: 9 Viability (intervention): NR	NR	EN + synbiotics (4 types of probiotics + 4 types of unspecified prebiotics) for 21 days	EN + 4 types of prebiotics
8	Klarin 2005 [37]	Critically ill patients on antibiotics n = 17	C.Random: no ITT: no Blinding: no Score: 6 Viability (intervention): NR	Mixed in fermented oatmeal, given via NG tube	EN + *Lactobacillus plantarum* 299v, 10^9^/day 50 ml every 6 h × 3 days then 25 ml every 6 h until ICU discharge	EN (Impact or Nutrodrip fiber). Some patients needed PN
9	McNaught 2005 [38]	ICU patients on antibiotics n = 130	C.Random: no ITT: yes Blinding: no Score: 7 Viability (intervention): NR	Oral, NJ tube	EN or PN + Proviva, (oatmeal and fruit drink) 5 × 10^7^ CFU/ml of *L. plantarum* 299 v × 500 mls until hospital discharge or beyond	EN or PN alone
10	Kotzampassi 2006 [39]	Patients with multiple trauma from 5 ICUs n = 77	C.Random: no ITT: no Blinding: double Score: 8 Viability (intervention): NR VAP determination: clinical	Endoscopic gastrostomy or NG tube	EN or PN + Synbiotic 2000 Forte 10^11^, 1 sachet/day for 15 days until ICU discharge	EN or PN + placebo (maltodextrin), mixed in tap water
11	Alberda 2007 [40]	ICU patients n = 28	C.Random: no ITT: yes; Blinding: double Score: 10 Viability (intervention): No for VSL # 3; Yes for bacteria sonicates	NG tube	Jevity Plus (EN) (10 g fructooligosaccharides/1000 mL and 12 g of soluble and insoluble fiber blend) + VSL # 3, 1 package BID, 9 × 10^11^ /day for 7 days until ICU discharge or EN discontinuation	Jevity Plus + placebo
12	Li 2007 [41]	Patients with severe acute pancreatitis n = 25	C.Random: no ITT: yes Blinding: no Score: 7 Viability (intervention): NR	Given enterally	Jinshuangqi (*bifidobacteria, lactobacillus and streptococcus*) 2.0 g TID on basis of traditional treatment Duration: NR	Traditional treatment
13	Olah 2007 [42]	Patients with severe acute pancreatitis n = 83	C.Random: no ITT: no Blinding: no Score: 9 Viability (intervention): NR	NJ tube	EN (Nutricion fiber) + Synbiotic 2000, 4 × 10^10^ CFU for 7 days	EN (Nutricion fiber) + 10 g plant fibers ((2.5 g each of Betaglucan, inulin, pectin and resistant starch) (prebiotics) BID for at least 2 days
14	Forestier 2008 [44]	Mixed ICU patients, 50 % on antibiotics n = 208	C.Random: not sure ITT: no Blinding: double Score: 8 Viability (intervention): NR VAP determination: objective	NG tube or oral (after tube removal)	*Lactobacillus casei rhamnosum*, 10^9^ CFU BID until ICU discharge	Placebo (growth medium never exposed to bacteria).
15	Besselink 2008 [43]	Patients with severe acute pancreatitis from 15 ICUs n = 298	C.Random: not sure ITT: yes Blinding: double Score:11 Viability (intervention): NR VAP determination: clinical	NJ tube or oral	EN (Nutrison multifiber) + Ecologic 641 10^10^ CFU BID for 28 days	EN (Nutrison multifiber) + placebo (cornstarch + maltodextrins)
16	Klarin 2008 [45]	ICU patients from 5 ICUs, on antibiotics for *C. Difficile* n = 68	C.Random: yes ITT: no Blinding: double Score: 10 Viability (intervention): NR	Mixed in fermented oatmeal added to enteral feeds NG tube	299 *Lactobacillus plantarum,* 8 × 10^8^ CFU/ml given as 6 × 100 ml doses every 12 h and after 50 ml given BID until ICU discharge	Same oatmeal gruel mixed with lactic acid
17	Knight 2009 [46]	General ICU patients n = 300	C.Random: yes ITT: no Blinding: double Score: 10 Viability (intervention): NR VAP determination: clinical	NJ or OG (orogastric) tube	EN (Nutrition Energy) + Synbiotic 2000 FORTE 4 × 10^11^ species/sachet BID for 28 days or ICU discharge	EN (Nutrison Energy) + Placebo
18	Barraud 2010 [47]	Mechanically ventilated ICU patients, 80 % on antibiotics n = 167	C.Random: yes ITT: yes; Blinding: double Score: 12 Viability (intervention): NR VAP determination: objective	NG tube	EN (Fresubin) + Ergyphilus 2 × 10^10^ per capsule + potato starch 5 capsules/day for 28 days	EN (fresubin) + placebo capsules (excipient of potato starch)
19	Morrow 2010 [10]	ICU patients n = 146	C.Random: no; ITT: yes; Blinding: double; Score:10 Viability (intervention): yes VAP determination: objective	Oropharynx and NG tube	EN (routine care) + *Lactobacillus rhamnosus* GG, 2 × 10^9^ BID as lubricant and mixed with water until extubation	EN (routine care) + inert plant starch inulin (prebiotic) BID as lubricant and mixed with water
20	Frohmader 2010 [48]	General ICU patients on antibiotics n = 45	C.Random: yes ITT: yes Blinding: double Score: 11 Viability (intervention): yes	NG or NJ tube	EN (Standard) + VSL #3 mixed in nutritional supplement (Sustagen), BID until hospital discharge	EN (Standard) + placebo mixed in nutritional supplement (Sustagen), BID
21	Ferrie 2011 [49]	Critically ill patients with diarrhea, n = 36	C.Random: no ITT: yes Blinding: double Score: 10 Viability (intervention): yes	NG tube	EN (Standard) + Culturelle (*Lactobacillus rhamnosus GG*)*,* 10^10^ species/capsule +280 mg inulin powder for 7 days	EN (Standard) + Raftiline, gelatin capsule with 280 mg inulin powder (prebiotic)
22	Sharma 2011 [50]	Patients with acute pancreatitis n = 50	C.Random: yes ITT: yes Blinding: double Score: 11 Viability (intervention): yes	Oral, NJ or NG	EN (standard) or oral 4 sachets each 2.5 × 10^9^ *Lactobacillus acidophilus, Bifidobacterium longus*, *Bifidobacterium bifidum & Bifidobacterium infantalis* + 25 gms fructose for 7 days	EN (Standard) + placebo
23	Tan 2011 [51]	Patients with closed head injury n = 52	C.Random: yes ITT: yes Blinding: single Score: 10 Viability (intervention): yes VAP determination: clinical	NG tube	EN (standard) total of 10^9^ bacteria i.e., 7 sachets each 0.5 × 10^8^ *Bifidobacterium longum,* 0.5 × 10^7^1 *Lactobacillus bulgaricus* and 0.5 × 10^7^ *Streptococcus thermophilus* for 21 days	EN (standard)
24	Cui 2013 [20]	Patients with severe acute pancreatitis n = 70	C.Random: no ITT: yes Blinding: no Score: 9 Viability (intervention): yes	EN	EN + bifidobacterium, 4 capsules (each 210 mg, 2.604 × 10^9^) every 12 h, given through nasal gastric tube. Total dose per day 20.832 × 10^9^	EN
25	Tan 2013 [21]	Severe craniocerebral trauma n = 52	C.Random: no ITT: other Blinding: no Score: 11 Viability (intervention): yes	NG tube	EN + 1 × 10^9^ bacteria of viable probiotics (Golden Bifid, 3.5 g 3 times per day) per day for 21 days.	EN (standard)
26	Wang 2013 [22]	Severe acute pancreatitis with intestinal ileus or abdominal distention. n = 183	C.Random: no ITT: yes Blinding: no Score: 6 Viability (intervention): NR	SBFT	EN (standard) + capsules 0.5 g TID containing *Bacillus subtilis* and *Enterococcus faecium* (5.0 × 10^7^Bacillus subtilis and 4.5 × 10^8^ *Enterococcus faecium* per 250 g capsule). Unclear timeframe.	EN (standard)
27	Lopez de Toro 2014 [23]	Medical and surgical ICU patients with multi-organ failure n = 89	C.Random: yes ITT: yes Blinding: no Score: 11 Viability (intervention): NR	EN	EN + symbiotic drink with streptococcus *Thermophilus, lactobacillus bulgaricus*, *Lactobacillus casei*, *lactobacillus acidophilus*, *bifidobacterium*, *Escherichia coli*, *coliformes* × 7 days (max 4.8 × 10^9^ UFC/ml).	EN and PN
28	Sanaie 2014 [24]	Critically ill pts, SIRS, expected length of stay ≥7 days n = 40	C.Random: yes ITT: yes Blinding: double Score: 9 Viability (intervention): yes	NG tube	EN (standard) + 2 sachets VSL#3 BID × 7 days.	EN (standard) + placebo
29	Rongrungruang 2015 [25]	Critically ill patients, expected to receive mechanical ventilation at least 72 h and had no VAP at enrollment n = 150	C.Random: no ITT: no Blinding: no Score: 6 Viability (intervention): yes	EN	80 ml of 8 × 10^9^ cfu of *Lactobacillus casei* (Shirota strain) (Yakult) for oral care after the standard oral care once daily An additional 80 ml of the product was given via enteral feeding once daily for 28 days or when their endotracheal tubes were removed	EN (standard) + oral care with 2 % chlorhexidine solution 4 times per day
30	Zeng 2016 [26]	Critically ill patients, expected to receive mechanical ventilation at least 48 h n = 235	C.Random: yes ITT: no Blinding: no Score: 5 Viability (intervention): yes	NG tube	1 capsule (Medilac-S, China) 0.5 g three times daily. Each probiotic capsule contained active *Bacillus subtilis* and *Enterococcus faecalis* at a concentration of 4.5 × 10^9^ /0.25 g and 0.5 × 10^9^/0.25 g, respectively	EN (standard)

*CFU* colony forming units, *C.Random* concealed randomization, *EN* enteral nutrition, *FOS* fructooligosaccharides, *NG* nasogastric, *NJ* nasojejunal, *NR* not reported, *OG* orogastric, *ITT* intention to treat, *SIRS*, systemic inflammatory response syndrome, *VAP* ventilator-associated pneumonia, *BID* twice daily. Trevis™: 1 capsule = *Lactobacillus acidophilus La5, Bifidobacterium lactis Bb12, Streptococcus thermophilus, Lactobacillus bulgaricus, 4* × *10* ^*9*^ */total*; Synbiotic 2000 Forte: 10^11^ CFU each of *Pediococcus pentoseceus 5-33:3, Leuconostoc mesenteroides 32-77:1, L. paracasei ssp paracasei 19, L. plantarum* 2362, and 2.5 g each of inulin, oat bran, pectin and resistant starch; Ergyphilus: 10^10^ *Lactobaccilus rhamnosus GG, Lactobacillus casei, L. acidophilus, Bifidobacterium bifidus*; VSL # 3: >10^10^ *Bifidobacterium longum, Bifidobacterium breve, >10* ^*10/g*^ *Bifidobacterium infantis, >10* ^*11/g*^ *L. acidophulus, L. plantarum, L. casei, L. bulgaris*, *and Streptococcus thermophiles*; Jinshuangqi: *B. longum >10* ^*7 CFU*^ *, L. bulgaricus >10* ^*6 CFU*^, and *S. Thermophilus >10* ^*6 CFU*^; Ecologic 641: *L. acidophilus, Lactobacillus salivarius, Lactococcus lactis, B. bifidus*, and *Bifidobacterium lactis*; Synbiotic 2000: *10* ^*10*^ *CFU each* of *P. pentoseceus 5-33:3, Leuconostoc mesenteroides 32-77:1, L. paracasei ssp paracasei 19, L. plantarum 2362*, and 2.5 g each of betaglucan, inulin, pectin and resistant starch; Golden Bifid: *B. bifidum, L. bulgaricus*, *and S. thermophilus triple*-*human probiotic-supplemented oligosaccharides FOS (bifidus factor)*

**Table 2:** Reported clinical outcomes in RCTs evaluating probiotics in critically ill patients

	Study	Mortality		Infections		Length of stay		Diarrhea	
		Intervention	Control	Intervention	Control	Intervention	Control	Intervention	Control
1	Tempe 1983 [30]	3/20 (15)	3/20 (15)	NR	NR	NR	NR	Diarrhea days 34/389 (9)	Diarrhea days 63/373 (17)
2	Schlotterer 1987 [31]	NR	NR	NR	NR	NR	NR	Diarrhea days 3/150 (2)	Diarrhea days 19/143 (13)
3	Heimburger 1994 [32]	NR	NR	NR	NR	NR	NR	Diarrhea 5/16 (31)	Diarrhea 2/18 (11)
4	Bleichner 1997 [33]	NR	NR	NR	NR	NR	NR	Diarrhea 18/64 (28) Days w/diarrhea 91/648 (14)	Diarrhea 24/64 (38) Days w/diarrhea 134/683 (20)
5	Kecskes 2003 [34]	Hospital 1/22 (5)	Hospital 2/23 (9)	Septic compl 1/22 (5)	Septic compl 7/23 (30)	Hospital 13.7 ± 8.7	Hospital 21.4 ± 17.9	NR	NR
6	Jain 2004 [36]	Hospital 22/45 (49)	Hospital 20/45 (45)	Septic compl 33/45 (73)	Septic compl 26/45 (58)	Hospital 24.0 ± 31.5 ICU 11.9 ± 13.1	Hospital 18.7 ± 13.5 ICU 9.0 ± 8.9	NR	NR
7	Lu 2004 [35]	Hospital 2/20 (10)	Hospital 1/20 (5)	Infectious compl 8/20 (40)	Infectious compl 11/20 (55)	NR	NR	NR	NR
8	Klarin 2005 [37]	Hospital 2/8 (25) ICU 1/8 (12)	Hospital 2/7 (29) ICU 2/7 (29)	NR	NR	Hospital 48.3 ± 30.4 ICU 14.2 ± 10.6	Hospital 34.3 ± 15.4 ICU 16.3 ± 15.7	NR	NR
9	McNaught 2005 [38]	18/52 (35)	18/51 (35)	Septic morbidity 21/52 (40)	Septic morbidity 22/51 (43)	ICU 5 (2–9)	ICU 4 (2–7)	NR	NR
10	Kotzampassi 2006 [39]	ICU 5/35 (14)	ICU 9/30 (30)	Infections 22/35 (63) VAP 19/35 (54) Septic compl 17/35 (49) Central venous line infections 13/35 (37) Wound infections 6/35 (17) UTI 6/35 (17)	Infections 27/30 (90) VAP 24/30 (80) Septic compl 23/30 (77) Central venous line infections 20/30 (66) Wound infections 8/30 (26) UTI 13/30 (43)	ICU 27.7 ± 15.2	ICU 41.3 ± 20.5	Diarrhea 5/35 (14)	Diarrhea 10/30 (30)
11	Alberda 2007 [40]	ICU 1/10 (10)	ICU 1/9 (11)	NR	NR	NR	NR	Diarrhea 1/10 (14)	Diarrhea 2/9 (23)
12	Li 2007 [41]	NR	NR	Infections 8/14 (58)	Infections 10/11 (91)	Hospital 42 ± 5.0	Hospital 49 ± 6.8	NR	NR
13	Olah 2007 [42]	Hospital 2/33 (6)	Hospital 6/29 (21)	Infections 9/33 (27) Septic compl 7/33 (12) Pancreatic abscess 2/33 (6) Infected pancreatic necrosis 2/33 (6) UTI 3/33 (9)	Infections 15/29 (52) Septic compl 17/29 (28) Pancreatic abscess 2/29 (7) Infected pancreatic necrosis 6/29 (21) UTI 3/33 (9)	Hospital 14.9 ± 3.3	Hospital 19.7 ± 4.5	NR	NR
14	Forestier 2008 [44]	NR	NR	VAP 19/102 (19)	VAP 21/106 (20)	ICU 22.5 ± 20.6	ICU 19.7 ± 16.7	NR	NR
15	Besselink 2008 [43]	24/152 (16)	9/144 (6)	Infections 46/152 (30) VAP 24/152 (16) Bacteremia 33/152 (22) Infected necrosis 21/152 (14) Urosepsis 1/52 (2)	Infections 41/144 (28) VAP 16/144 (11) Bacteremia 22/144 (15) Infected necrosis 14/144 (10) Urosepsis 2/144 (1)	Hospital 28.9 ± 41.5 ICU 6.6 ± 17	Hospital 23.5 ± 25.9 ICU 3.0 ± 9.3	Diarrhea 25/152 (16)	Diarrhea 28/144 (19)
16	Klarin 2008 [45]	Hospital 3/22 (5) ICU 2/22 (9)	Hospital 2/22 (0) ICU 2/22 (9)	*C. difficile* + fecal samples 0/71	*C. difficile* + fecal samples 4/80	Hospital 25.8 ± 19.4 ICU 8.0 ± 5.4	Hospital 50.3 ± 75.2 ICU 11.6 ± 14	NR	NR
17	Knight 2009 [46]	Hospital 35/130 (27) ICU 28/130 (22)	Hospital 42/129 (33) ICU 34/129 (26)	VAP 12/130 (9)	VAP 17/129 (13)	ICU 6 (3–11)	ICU 7 (3–14)	Diarrhea 7/130 (5)	Diarrhea 9/129 (7)
18	Barraud 2010 [47]	ICU 21/87 (24) 28 days 22/87 (25) 90 days 27/87 (31)	ICU 21/80 (26) 28 days 19/80 (24) 90 days 24/80 (30)	All infections 30/87 (34) Infection > 96 h 26/87 (30) VAP 23/87 (26) Catheter-related BSI 3/87 (4) UTI 4/87 (5)	All infections 30/80 (38) Infection > 96 h 29/80 (36) VAP 15/80 (19) Catheter-related BSI 11/80 (14) UTI 4/89 (5)	Hospital 26.6 ± 22.3 ICU 18.7 ± 12.4	Hospital 28.9 ± 26.4 ICU 20.2 ± 20.8	Diarrhea 48/87 (55)	Diarrhea 42/80 (53)
19	Morrow 2010 [10]	12/68 (18)	15/70 (21)	VAP 13/73 (18)	VAP 28/73 (38)	Hospital 21.4 ± 14.9 ICU 14.8 ± 11.8	Hospital 21.7 ± 17.4 ICU 14.6 ± 11.6	Non *C. difficile* diarrhea 42/68 (62) *C. difficile* diarrhea 4/68 (6)	Non *C. difficile* diarrhea 44/70 (63) *C. difficile* diarrhea 13/70 (19)
20	Frohmader 2010 [48]	5/20 (25)	3/25 (12)	NR	NR	ICU 7.3 ± 5.7	ICU 8.1 ± 4	Diarrhea episodes/pt/day 0.53 ± 0.54	Diarrhea episodes/pt/day 1.05 ± 1.08
21	Ferrie 2011 [49]	Hospital 2/18 (11) 6 months 7/18 (39)	Hospital 2/18 (11) 6 months 5/18 (28)	Infections 14/18 (78)	Infections 16/18 (89)	Hospital 54.50 ± 31.26 ICU 32.04 ± 24.46	Hospital 59.04 ± 33.92 ICU 29.75 ± 18.81	Duration of diarrhea 3.83 ± 2.39 Loose stools/day 1.58 ± 0.88	Duration of diarrhea 2.56 ± 1.85 Loose stools/day 1.10 ± 0.79
22	Sharma 2011 [50]	Hospital 2/24 (8)	Hospital 2/26 (8)	NR	NR	Hospital 13.23 ± 18.19 ICU 4.94 ± 9.54	Hospital 9.69 ± 9.69 ICU 4.0 ± 5.86	NR	NR
23	Tan 2011 [51]	28 days 3/26 (12)	28 days 5/26 (19)	Infections 9/26 (35) VAP 7/26 (27)	Infections 15/26 (58) VAP 13/26 (50)	ICU 6.8 ± 3.8	ICU 10.7 ± 7.3	NR	NR
24	Cui 2013 [20]	Hospital 1/23 (4)	Hospital 1/25 (4)	N/A	N/A	Hospital 10.4 ± 3.9 (23)	Hospital 13.4 ± 5.2 (25)	NR	NR
25	Tan 2013 [21]	28 days 23/26 (12)	28 days 5/26 (19)	NR	NR	ICU 6.8 ± 3.8 (26)	ICU 10.7 ± 7.3 (26)	NR	NR
26	Wang 2013 [22]	Unspecified 1/62 (8.1)	Unspecified 3/61 (9.8)	Pancreatic sepsis 8/62 (13) MODS 7/62 (11.3)	Pancreatic sepsis 13/61 (21) MODS 15/61 (25)	NR	NR	NR	NR
27	Lopez de Toro 2014 [23]	Hospital 19/46 (41) ICU 15/46 (33)	Hospital 18/43 (42) ICU 14/43 (33)	Hospital acquired infections 9/46 (20)	Hospital acquired infections 13/43 (30)	Hospital 18.5 (10–36) ICU 9 (3–19)	Hospital 24.5 (10–38) ICU 8 (2.5–16.5)	NR	NR
28	Sanaie 2014 [24]	28 days 2/20 (10)	28 days 5/20 (25)	Bacteremia 2/20(10)	Bacteremia 5/20(25)	ICU 13.85 ± 6.96	ICU 14.16 ± 5.97	NR	NR
29	Rongrungruang 2015 [25]	28 days 18/75 (24) 90 day 25/75 (33)	28 days 17/75 (22.7) 90 day 26/75 (34.7)	VAP 18/75 (24)	VAP 22/75 (29.3)	Hospital 20 (2–106) ICU 30.5 (4–98)	Hospital 19 (3–171) ICU 19 (5–30)	Diarrhea 19/75 (25.3)	Diarrhea 14/75 (18.7)
30	Zeng 2016 [26]	Hospital 26/118 (22) ICU 15/118 (12.7)	Hospital 25/117 (21.4) ICU 9/117 (7.7)	VAP 43/118 (36.4)	VAP 59/117 (50.4)	Hospital 13.5 ± 12.4 ICU 18 (14–32)	Hospital 10.6 ± 10.2 ICU 22 (11–56)	NR	NR

*BSI* blood stream infection, *ICU* intensive care unit, *NR* not reported, *RCT* randomized control trial, *UTI* urinary tract infection, *VAP* ventilator associated pneumonia, *N/A* non-attributable, *compl* complications, *MODS* Multiple Organ Dysfunction Syndrome, Days w/ diarrhea - days with diarrhea

### Primary outcome: infections

#### Overall effect on new infections

**Fig. 1:**
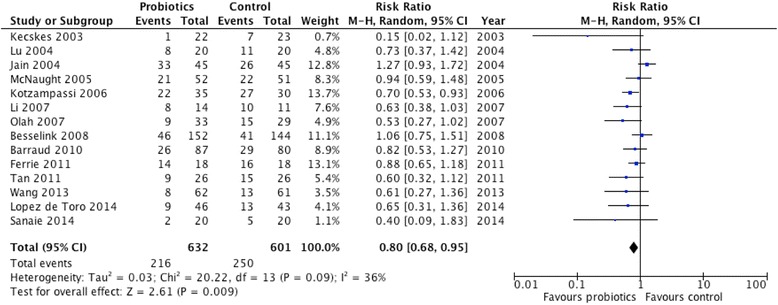
Effect of probiotics on overall infections (n = 14). *CI* confidence interval, *M-H* Mantel-Haenszel test

Aggregating the results of the 14 trials reporting overall infections, probiotics were associated with a significant reduction in infections (RR 0.80, 95 % CI 0.68, 0.95, *P* = 0.009; heterogeneity *I*^2^ = 36 %, *P* = 0.09; Fig. 1).

### Secondary outcomes

#### Ventilator associated pneumonia

**Fig. 2:**
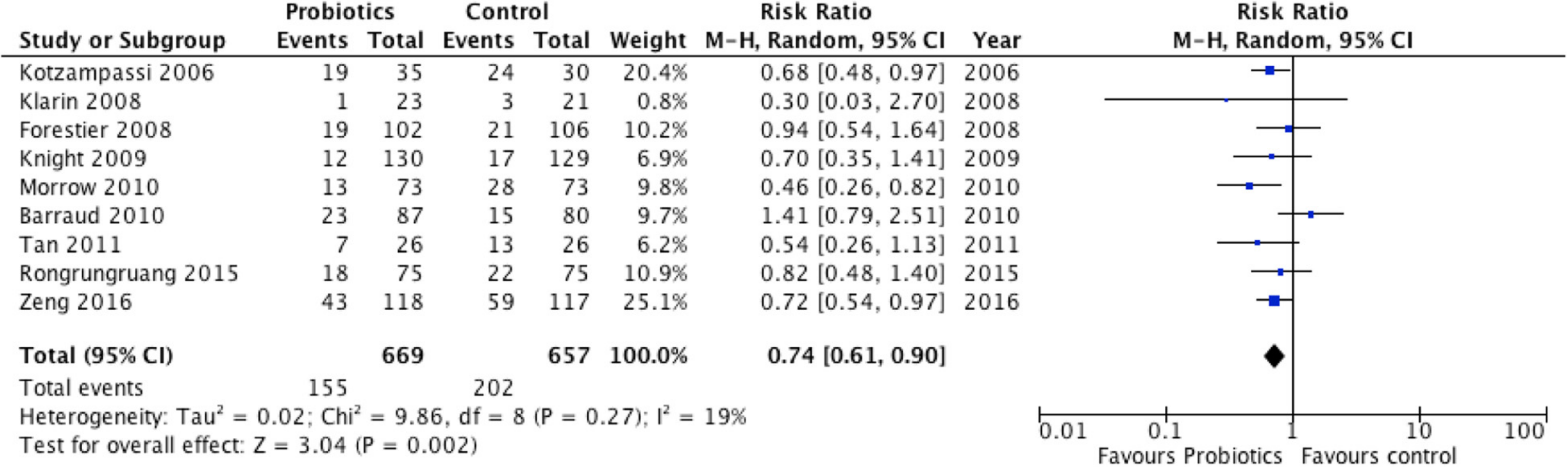
Effects of probiotics therapy on the incidence of ventilator-associated pneumonia (n = 9). CI confidence interval, *M-H* Mantel-Haenszel test

Aggregating the data from 9 trials that reported VAP, there was a significant reduction in the incidence of VAP (RR 0.74, 95 % CI 0.61, 0.90, *P* = 0.002; I^2^ = 19 %, *P* = 0.27; Fig. 2).

#### Overall effect on mortality

**Fig. 3:**
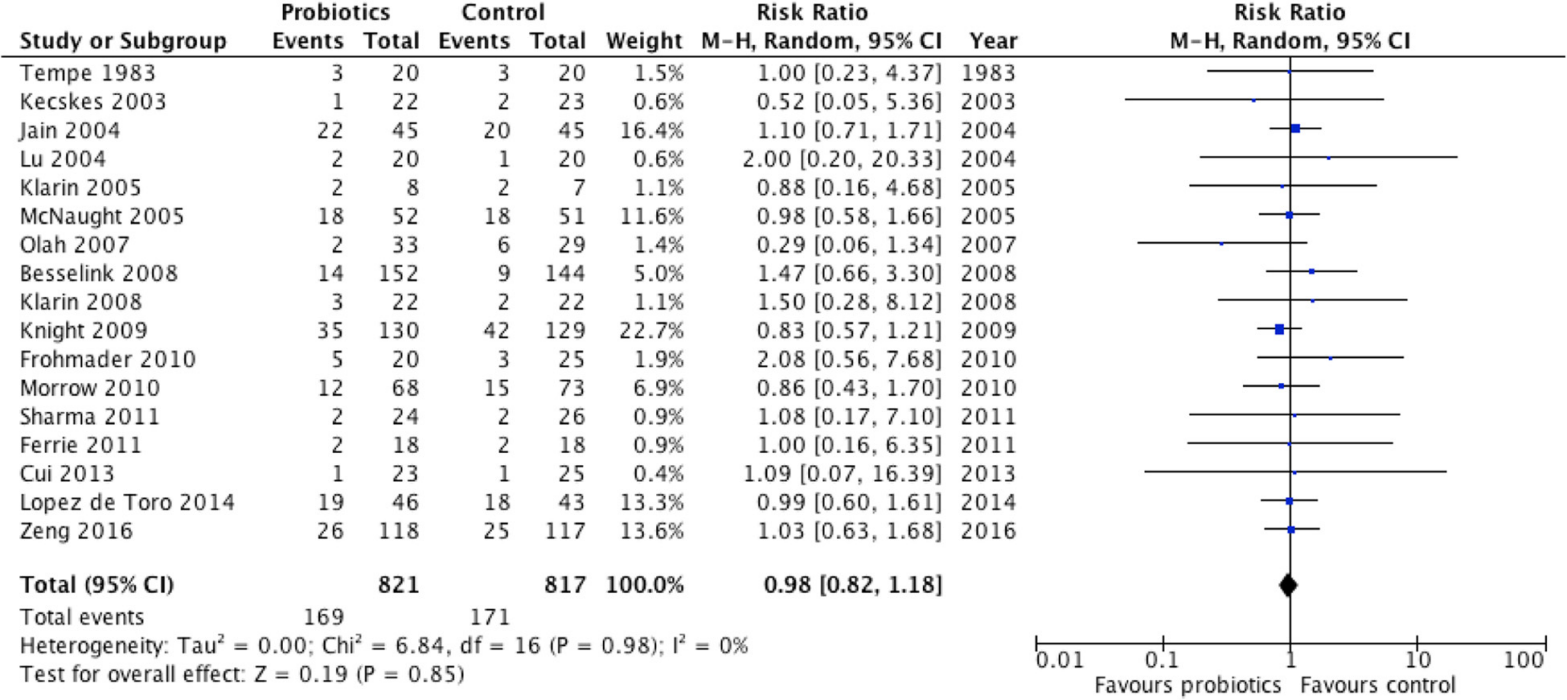
Effect on hospital mortality (n = 17). *CI* confidence interval, *M-H* Mantel-Haenszel test

A total of 17 studies reported on hospital mortality. When statistically aggregated, probiotic therapy did not significantly affect mortality (RR 0.98, 95 % CI 0.82, 1.18, *P* = 0.85; *I*^2^ = 0 %, Fig. 3). Moreover, probiotics did not show any effect on ICU mortality (RR 0.90, 95 % CI 0.70, 1.17, *P* = 0.44; *I*^2^ = 0 %).

#### Overall effect on ICU length of stay

Aggregating the data from the 14 RCTs reporting on ICU LOS, there were no significant differences between the groups (weighted mean difference (WMD) -3.26, 95 % CI -7.82, 1.31, *P* = 0.16; *I*^2^ = 93 %, *P* < 0.00001).

#### Overall effect on hospital length of stay

Aggregating the data from the nine RCTs that reported hospital LOS, there were no significant differences between the groups (WMD -0.58, 95 % CI -3.66, 2.50, *P* = 0.71; *I*^2^ = 74 %, *P* < 0.00001).

#### Diarrhea

**Fig. 4:**
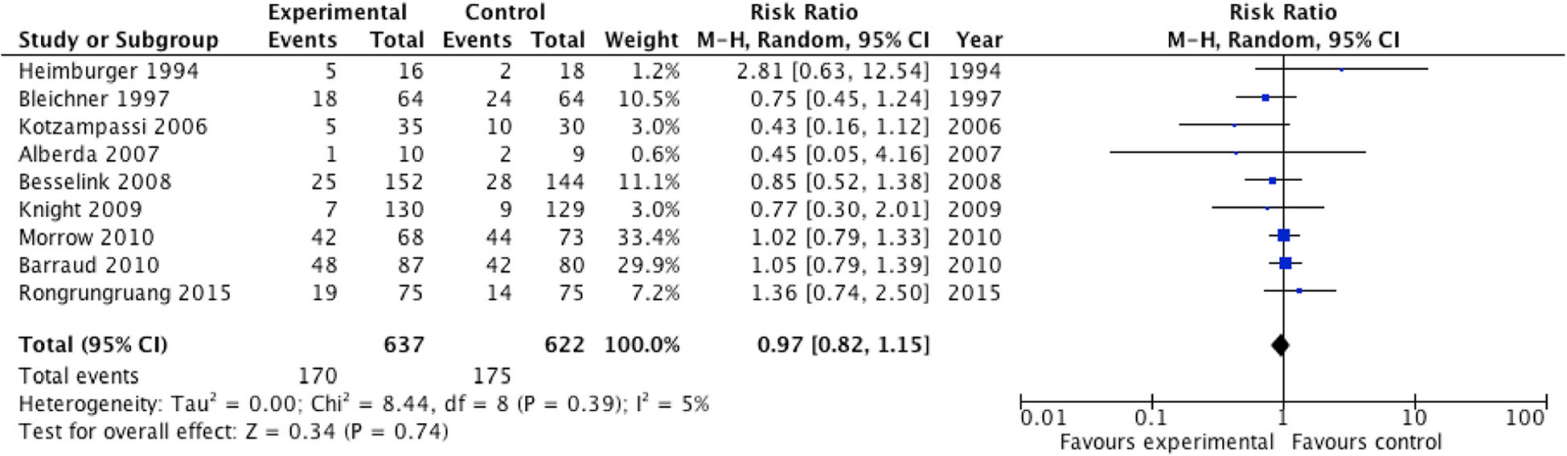
Effect of probiotics on diarrhea (n = 9). *CI* confidence interval, *M-H* Mantel-Haenszel test

Aggregating the data from nine trials that reported on diarrhea, probiotics had no effect (RR 0.97; 95 % CI 0.82, 1.15; *P* = 0.74; *I*^2^ 5 %, *P* = 0.39; Fig. 4).

#### Antibiotic days

**Fig. 5:**
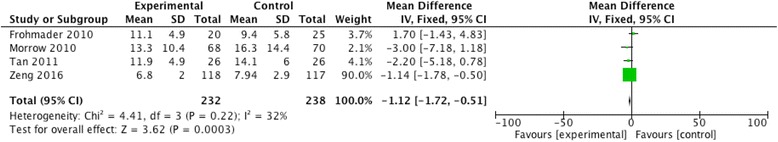
Effect of probiotics on antibiotic days (n = 4). *CI* confidence interval; *WMD* weighted mean difference

When we aggregated the data of four trials reporting on antibiotic days, we found that probiotics were significantly associated with a reduction in the duration of antibiotic therapy (WMD -1.12, 95 % CI -1.72, -0.51, *P* = 0.0003; *I*^2^ = 32 %, *P* = 0.22; Fig. 5).

### Subgroup analysis

#### Probiotics daily dose

There were similar rates of infectious complications in RCTs using high-dose probiotic therapy (n = 8 trials) (0.87; 95 % CI 0.72–1.06; *P* =0.18; *I*^2^ = 43 %) and in those using lower daily doses (n = 2 trials) RR 0.40; 95 % CI 0.11–1.50; *P* = 0.18; *I*^2^ = 48 %. The difference between subgroups was not significant, *P* = 0.25).

#### L. plantarum vs. non L. plantarum

Subgroup analyses showed that four trials administering *L. plantarum*, either alone or in combination with other probiotics, were associated with a significant reduction in overall infections (RR = 0.70, 95 % CI 0.50, 0.97; *P* = 0.03; *I*^2^ = 36 %). However, in the 10 trials that did not include *L. plantarum*, there was no significant effect on overall infectious complications (RR = 0.88, 95 % CI 0.74,1.04; *P* = 0.15; *I*^2^ = 21 %). Test for subgroup differences between groups was not significant (*P* = 0.21).

#### L. rhamnosus GG vs. other probiotics

In two trials using LGG there was no significant effect on reduction in infectious complications (RR 0.86; 95 % CI 0.67–1.10; *P* = 0.22; *I*^2^ = 0 %). However, in 12 trials that supplemented other probiotics there was a significant reduction in overall infections (RR 0.77; 95 % CI 0.62–0.95; *P* = *I*^2^ = 45 %); *P* = 0.52 for the difference between groups.

#### Synbiotics vs. other strategies

**Fig. 6:**
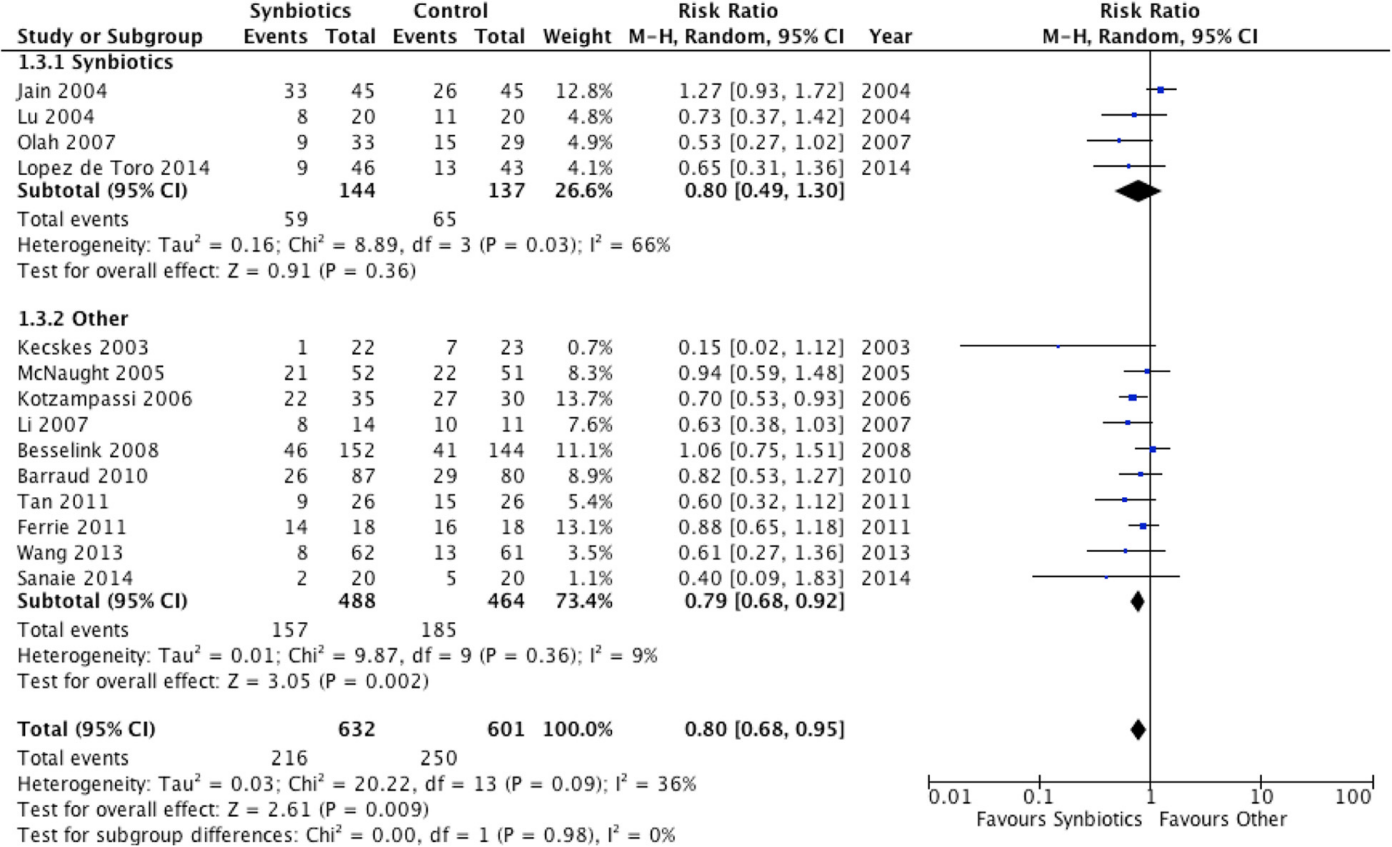
Subgroup analysis: effect on infections of synbiotics (n = 4) versus probiotics alone (n = 10). *CI* confidence interval, *M-H* Mantel-Haenszel test

In subgroup analyses of the four trials that administered synbiotics there was no effect on infections (RR = 0.80, 95 % CI 0.49, 1.30, *P* = 0.36; *I*^2^ = 66 %, *P* = 0.03) (Fig. 6). However, in 10 studies that administered probiotics alone there was a significant reduction in the incidence of infections (RR 0.79, 95 % CI 0.68, 0.92, *P* = 0.002; *I*^2^ = 9 %, *P* = 0.36). The P value for subgroup differences was not significant (P = 0.98). (Fig. 6).

#### Higher vs. lower mortality

**Fig. 7:**
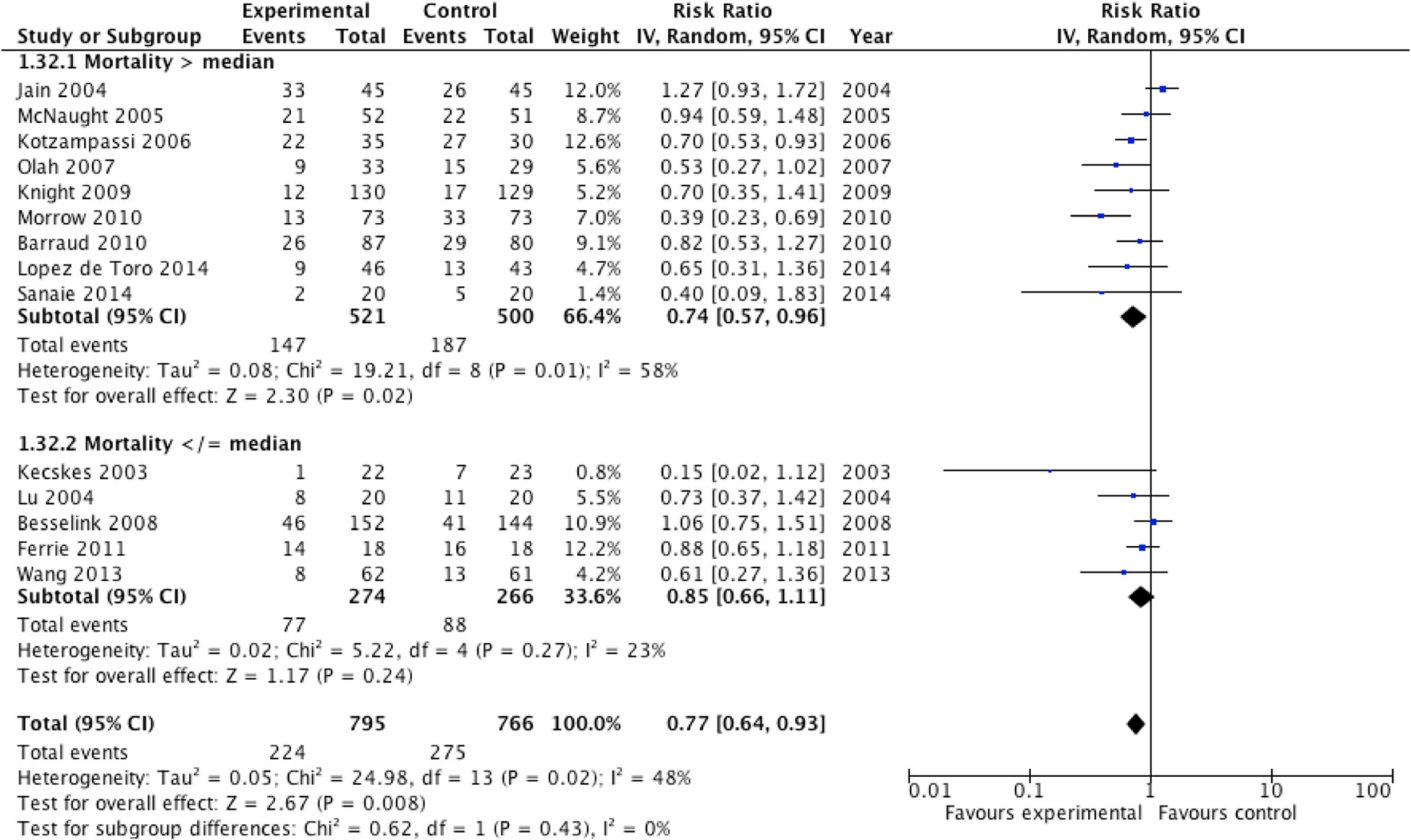
Subgroup analysis: effect of higher (n = 9) vs. lower mortality (n = 5) on infections. CI confidence interval, *IV* inverse variance

The median hospital mortality rate of all the trials (or ICU mortality when hospital mortality was not reported) in the control group was 19 %. After aggregating nine studies with a higher mortality rate, probiotics significantly reduced the incidence of infections (RR 0.74; 95 % CI 0.57, 0.96; *P* = 0.02; *I*^2^ = 58 %, *P* = 0.01) (Fig. 7). However, probiotics did not have an effect on infections in the five studies with lower mortality (RR 0.85; 95 % CI 0.66, 1.11; *P* = 0.24; *I*^2^ = 23 %, *P* = 0.27). The test for subgroup differences was not significant (*P* = 0.43) (Fig. 7).

#### Higher vs. lower methodological score

**Fig. 8:**
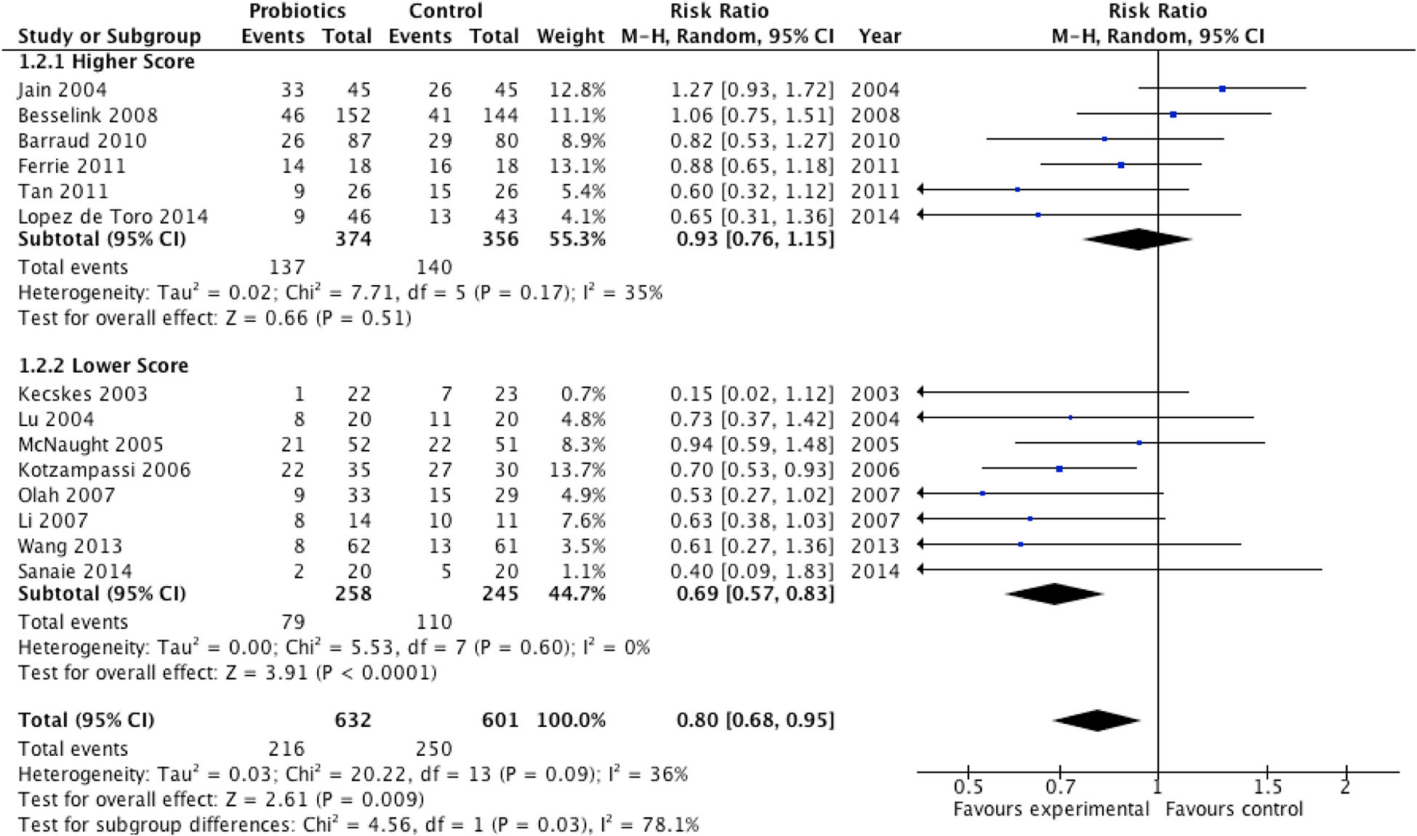
Subgroup analysis: effect of higher (n = 6) vs. lower methodological scores (n = 8) on infections. *CI* confidence interval, *M-H* Mantel-Haenszel test

The median methodological score was 9.5. In six trials with a higher score (≥9.5) there was no effect on infections (RR 0.93; 95 % CI 0.76, 1.15; *P* = 0.51; *I*^2^ = 35 %, *P* = 0.17), whereas in eight trials with a lower methodological score (<9.5) there was a significant reduction in infectious complications (RR 0.69, 95 % CI 0.57, 0.83, *P* < 0.0001; *I*^2^ = 0 %) (Fig. 8); the overall test for subgroup differences was significant for these subgroups (*P* = 0.03).

### Publication bias

**Fig. 9:**
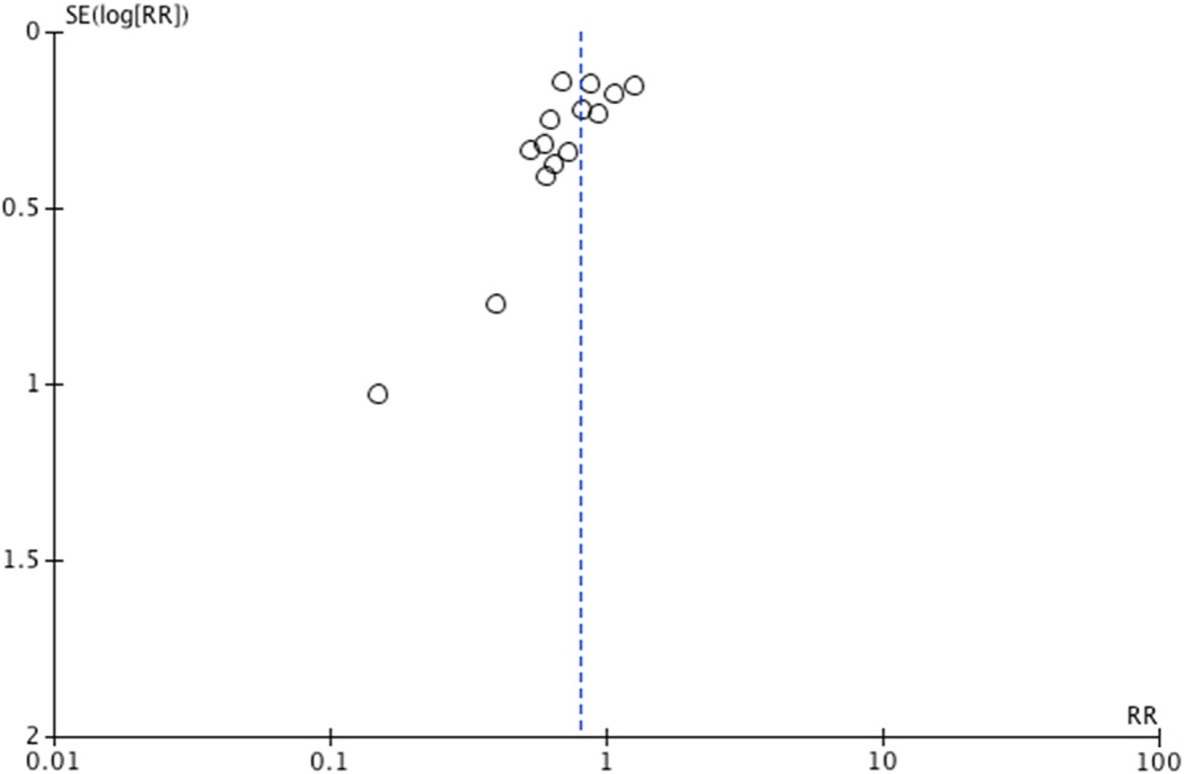
Funnel plot of primary outcome (n = 14). Overall new infections (test for asymmetry, OR -2.30, 95 % CI -3.56, -1.05, *P* = 0.001). *OR* odds ratio

There was indication that potential publication bias influenced the observed aggregated results. In fact, funnel plots were created for each study outcome and the tests of asymmetry were significant for overall new infections (odds ratio (OR) -2.30, 95 % CI -3.56, -1.05, *P* = 0.001; Fig. 9) and hospital LOS (OR -3.32, 95 % CI -6.12, -0.52, *P* = 0.024). However, the test for asymmetry was not significant for any other outcome (VAP, *P* = 0.76; mortality, *P* = 0.80; ICU LOS, *P* = 0.47; diarrhea, *P* = 0.18).

## Discussion

To date, our systematic review and meta-analysis is the largest and most updated evaluation of the overall effects of probiotics in the critically ill. It is also the first to include an analysis of symbiotic (probiotic/fiber combinations). Based on the analysis of 30 trials enrolling 2972 patients we demonstrated that probiotics are associated with a significant reduction in ICU-acquired infections, including VAP, which is the most common infectious complication in the critically ill. This significant effect on VAP is a new finding from our previous systematic reviews. Further, the beneficial effect of probiotics on reduction of infections is stronger with the publication of the new trials and the data no longer show a statistically significant effect of heterogeneity on this endpoint. Despite the probiotic effect of reducing infectious complications, this therapy did not influence ICU or hospital mortality, although none of the trials were powered to detect an effect on mortality. Overall, there was a tendency towards a reduction in ICU LOS and probiotic therapy did not influence other clinical endpoints such as hospital LOS, and diarrhea. Statistical and clinical heterogeneity was observed for some endpoints, although this was significant for the key endpoints of infectious complications and VAP. In addition, publication bias for overall infections and hospital LOS means that larger, well-powered, and more definitive clinical trials are urgently needed aimed to avoid these biases. Moreover, subgroup analysis showed that those trials with lower methodological quality exhibit the best treatment effect, which is another issue indicating that larger, well-designed studies are needed. Again, with the exception of four trials, most of the included studies (n = 14) that reported mortality had small sample sizes, and hence were underpowered and inadequate to detect any clinically important treatment effects of probiotic therapy on mortality. Moreover, the inferences we can make from our current findings are further weakened, as randomization was concealed in 30 % of trials, whereas double-blinding was performed in 67 % of trials.

Over recent years, several systematic reviews and meta-analyses have been conducted, although our meta-analysis is the largest and most current to date, as it contains the seven new suitable trials published since the most recent comprehensive meta-analysis publication on this topic, which focused on overall infections and other outcomes (not primarily VAP) in 2012 [14]. Further, these previous systematic reviews did not include analysis of synbiotic therapy. Overall, we have examined several relevant clinical outcomes in a heterogenous ICU patient population, and therefore our results could be applied to a broad group of critically ill patients with sepsis, trauma, severe pancreatitis, or who have undergone surgery. Specific to pancreatitis, concerns have been raised about the safety of probiotic therapy following the 2008 trial, Probiotic prophylaxis in patients with predicted severe acute pancreatitis (PROPATRIA) [43], which showed that Ecologic 641® given with fiber post-pyloric was associated with higher mortality and bowel ischemia. This post-pyloric method of administration was associated with an increase in small bowel necrosis, which was subsequently associated with death in a number of patients receiving the prebiotic fiber/probiotic mixture. It is possible that the post-pyloric administration of this fiber/multiple probiotic strain mixture in patients with pancreatitis may carry significant risk and should likely be avoided [52]. Unfortunately, there were significant ethical and statistical concerns raised about the conduct of the trial [53], limiting the utility of the data. Further, more recently, a systematic review and meta-analysis by Gou et al. [54] found that probiotics had neither beneficial nor adverse effects in patients with pancreatitis.

Despite the limitations of the PROPRIATA trial, it has contributed to concerns around the safety of probiotic administration in critical illness and limited the design of larger-scale clinical trials and/or more routine clinical administration of live probiotics. To address this, the American Health Care Research and Quality (AHRQ) agency reviewed and reported on the safety of probiotic therapy in over 600 published clinical trials and case reports [55]. It should be reassuring to future investigators that the overall conclusion of the extensive AHRQ report indicates that probiotic therapy in both adults and pediatric populations was not been found to be associated with any increased risk of infectious or other adverse events in either healthy or ill patients. Importantly, their report revealed a trend towards *less* adverse events in probiotic-treated critically ill patients, although isolated adverse effects of probiotic administration have been reported [56]. In any case, careful and appropriate safety monitoring in all future probiotic clinical trials should be conducted.

Recent data indicate that infection during critical illness continues to be a major challenge worldwide. A multi-national ICU study of 14,414 patients in 1265 ICUs from 75 countries, revealed that 51 % of ICU patients were considered infected on the day of survey and 71 % were receiving antibiotics [57]. Of the infections in this study, 64 % were of respiratory origin and the ICU mortality rate in infected patients was more than twice that of non-infected patients (25 % vs. 11 %, *P* < 0.001), as was the hospital mortality rate (33 % infected vs. 15 % non-infected, *P* < 0.001) [57].

Currently, VAP is the second most common nosocomial infection in the USA and the most prevalent ICU-acquired infection. Notwithstanding, its incidence is highly variable depending on diagnostic criteria used to identify this infectious complication. In fact, in 2015 Ego et al. [58] reported that the incidence of VAP ranged from 4 % to 42 % when using the six published sets of criteria and from 0 % to 44 % when using the 89 combinations of criteria for hypoxemia, inflammatory response, bronchitis, chest radiography, and microbiologic findings. In our systematic review we found that the incidence of VAP ranged from 9 % [46] to 80 % [39]. Additionally, the apparent effect of probiotics on VAP is largely driven by the studies of Kotzampassi et al. [39] study and the Zeng et al. [26]; both trials explain 45.5 % of the signal and thus, provide an unstable estimate. Moreover, current knowledge shows that VAP is associated with high cost and poor clinical outcomes [59]. In 2002, Rello et al. [60] demonstrated that VAP leads to an additional US$40,000 in hospital charges per patient, and recently it has been suggested that the use of prophylactic probiotics may be cost-effective for prevention of VAP from a hospital perspective [61].

Probiotic therapy may prevent VAP and other infections by restoring non-pathogenic flora, which competes with nosocomial pathogens inhibiting their overgrowth, modulating local and systemic immune response, and improving gut barrier function. However, in spite of these protective effects the role of probiotics as a non-pharmacological strategy in preventing VAP has previously been inconclusive. In 2010, Siempos et al. [12] aggregated five probiotic trials demonstrating a reduction in the incidence of VAP, whereas in 2012 Petrof et al. [14] and subsequently Barraud et al. [13] and Wang et al. [15] did not demonstrate any significant effect of probiotic therapy on VAP. More recently, a Cochrane review of probiotic therapy specifically for VAP [17], found with low quality of evidence that probiotic therapy is associated with a reduction in the incidence of VAP. Our current systematic review demonstrates a significant treatment effect of probiotics in reducing VAP and did not demonstrate statistical heterogeneity, strengthening the signal that this may be an effective therapy for VAP. Recently, a Canadian survey [27] on the use of probiotics as a prophylactic strategy for VAP showed that most Canadian ICU pharmacists have used probiotics at least once, although they do not routinely recommend probiotics for the prevention of VAP.

Currently, a large number of clinical trials have demonstrated that probiotics may reduce the incidence of antibiotic-associated diarrhea and *Clostridium difficile* infections, and systematic reviews have confirmed a significant signal of benefit on reduction of diarrhea and *C. difficile*-related colitis in all patients (not confined to ICU patients) [62, 63]. Our results, when focused on ICU patients do not currently demonstrate a treatment benefit of probiotics in preventing and treating diarrhea in the critically ill, including antibiotic-associated diarrhea.

An interesting finding of our meta-analysis was a reduction in antibiotic use in those patients who received probiotics. Nonetheless, only four trials [10, 21, 26, 48] comprising 13 % of included studies reported duration of antibiotic therapy as an outcome. In addition, the study of Zeng et al. contributed to 90 % of the signal, which is a very unstable estimate that weakens our finding. Therefore, probiotics may shorten the duration of antibiotic therapy, although the limited clinical trial data available for this endpoint limits the strength of these findings and further investigation of this effect is needed.

We currently have a greater understanding about the potential benefits of probiotics therapy in critical illness, although much more data are needed. Subgroup analysis found that certain strains such as *L. plantarum* alone or in combination was associated with a significant reduction in overall infections, although the test for subgroup differences was not significant (*P* = 0.21). Certain specific biological properties have been described for *L. plantarum*, including an ability to prevent adhesion of pathogens to the intestinal epithelium secondary to the production of adhesins, enolase, and phosphoglycerate kinase on the bacterial surface [64, 65]. These mechanisms may be crucial to reduction of bacterial translocation and modulation of local inflammatory response, and therefore the effect of this strain on systemic infectious complications. Interestingly, probiotics alone had a greater effect than synbiotics on infections, although the difference between these subgroups was not significant (*P* = 0.98) and more data on the specific effects of different prebiotic fibers are needed. Finally, future trials also need to focus on evaluating the changes in the microbiome following critical illness and the effect of probiotic or synbiotics on restoring a healthy microbiome in treated patients [66]. Recent advances in microbiome sequencing technology (16 s rRNA) in the last few years have resulted in an unprecedented growth in the amount of sequence data that can be collected at a previously unattainable low cost [66]. Thus, if we speculate that a specific probiotic or synbiotic therapy can be used to treat dysbiosis (a pathological change in the patient’s bacterial flora) and restore a healthy microbiome, we need to evaluate this with the new accessible microbiome analysis techniques currently available. This may help us target probiotic or probiotic mixtures in the future and increase the personalization of care.

The strength of this current systematic review includes the use of several methods to reduce bias (comprehensive literature search, duplicate data abstraction, specific criteria for searching and analysis), and the analysis of relevant clinical outcomes in the critically ill. However, several important limitations in drawing strong treatment inferences are present. These include the significant potential for publication bias for the infection and hospital LOS outcomes, and the small numbers of trials included in subgroup analyses. In addition, the variety of probiotic strains, wide range of daily doses, and length of administration of probiotic therapy among the different trials weaken any possible clinical conclusions and recommendations. We were also unable to perform subgroup analysis for all clinical outcomes due to the limited number of studies evaluating each endpoint.

Based on our current data, there is not currently sufficient evidence to make a final strong recommendation for probiotics to be utilized in the prevention of infections, including VAP, in the critically ill. However, our current guideline recommendations suggest that probiotics should be considered to improve outcome in critically ill patients [19]. Future trials continue to need to address questions about timing, daily dose, and duration of therapy, which still remain unanswered.

## Conclusion

In the largest systematic review and meta-analysis of probiotics to date, we demonstrated that in 30 trials enrolling 2972 patients, probiotics significantly reduced the incidence of infectious complications, including new episodes of VAP in critically ill patients. This finding is limited by clinical heterogeneity and potential publication bias for the overall infection outcome. This precludes a more meaningful statistical conclusion of the efficacy of probiotic therapy on overall infections and potentially the prevention of VAP in critical illness. Moreover, according to our findings probiotics has been demonstrated to be more effective in those trials with higher mortality in the control group. Probiotic therapy with *L. plantarum* currently demonstrates the most significant effect on the reduction of infections. Overall, the variety of strains, wide range of daily doses, and length of administration of probiotics weakens the strength of our conclusion. Certainly, additional large-scale, adequately powered, well-designed clinical trials, aimed at confirming our observations, are needed and warranted.

## Key messages

- Critical illness is characterized by a loss of commensal flora and an overgrowth of potentially pathogenic bacteria, leading to a high susceptibility of nosocomial infections
- Probiotics are living non-pathogenic microorganisms, which may protect the gut barrier, attenuate pathogen overgrowth, decrease bacterial translocation and prevent infection in ICU patients
- Probiotic use in the ICU remains widespread and controversial, current guidelines are not conclusive, and a significant number of new trials of probiotics have been published recently, which requires a current and comprehensive systematic analysis of probiotic and synbiotic therapy in critically ill patients
- Probiotics were associated with a significant reduction in infections and a significant reduction in the incidence of ventilator-associated pneumonia (VAP) was found in critically ill patients receiving probiotics alone versus synbiotic mixtures, demonstrating the greatest improvement in infectious outcome, limited synbiotic trial data are currently available
- Currently, clinical heterogeneity and potential publication bias reduce strong clinical recommendations and indicate further high-quality clinical trials are needed to conclusively prove these benefits
- Probiotics shows promise for the reduction of infections, including VAP in critical illness, and should be considered in critically ill patients

## Abbreviations

CFU, colony-forming unit; CI, confidence interval; C.Random, concealed randomization; EN, enteral nutrition; ICU, intensive care unit; Ig A, immunoglobulin A; ITT, intention to treat; LGG, *Lactobacillus rhamnosus* strain GG; LOS, length of stay; MV, mechanical ventilation; NA, non-attributable; NR, non-reported; OR, odds ratio; RCT, randomized controlled trial; RNA, ribonucleic acid; RR, risk ratio; VAP, ventilator-associated pneumonia; WMD, weighted mean difference
