# Erratum to: Probiotic and synbiotic therapy in critical illness: a systematic review and meta-analysis

**DOI:** 10.1186/s13054-017-1622-4

**Published:** 2017-02-27

**Authors:** William Manzanares, Paul E. Wischmeyer

**Affiliations:** 1 Department of Critical Care, Intensive Care Unit, Hospital de Clínicas (University Hospital), Faculty of Medicine, Universidad de la República (UdelaR), Italia Ave. 14th Floor. 11.600, Montevideo, Uruguay; 2 Department of Anesthesiology and Surgery, Duke University School of Medicine, Duke Clinical Research Institute, 2400 Pratt Street, Office: NP 7060, Durham, NC 27705 USA

## Erratum

Unfortunately, the original version of this article [1] published in Critical Care contained an error. We have made an error in the calculation of the pooled risk ratio (RR) and 95% confidence interval (CI) for the effect of probiotics and synbiotics on hospital mortality. Currently, the revised effect on overall mortality is 1.02 (95% CI 0.85,1.22; P = 0.83, I^2^ = 0%). The corrected figure is shown in this erratum (Fig. 1). This error has not modified the results and conclusions of the meta-analysis.

**Fig. 1:**
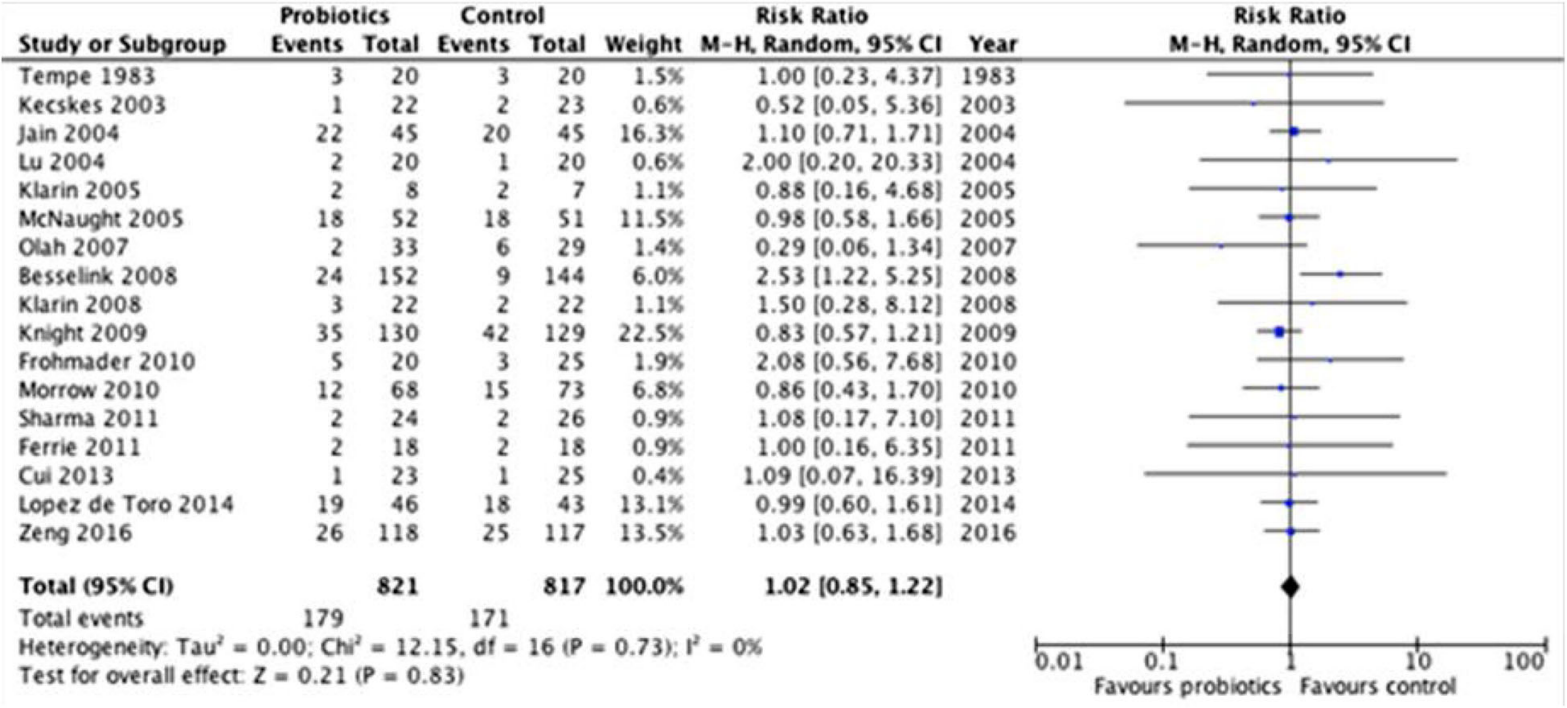
Effect of probiotic therapy on Mortality (n = 17). Original forest plot after reassesing the effect of probiotics on mortality
